# Clinical and imaging features preoperative evaluation of histological grade and microvascular infiltration of hepatocellular carcinoma

**DOI:** 10.1186/s12876-022-02449-w

**Published:** 2022-08-01

**Authors:** Ling Zhang, Jiong-bin Lin, Ming Jia, Chen-cai Zhang, Rong Xu, Le Guo, Xiao-jia Lin, Quan-shi Wang

**Affiliations:** 1 Department of Radiology, Nanfang Hospital, Southern Medical University, Guangzhou, 510515 China; 2 NanFang PET Center, Nanfang Hospital, Southern Medical University, 1838 Guangzhou Avenue North, Guangzhou, 510515 Guangdong China

**Keywords:** Hepatocellular carcinoma, Histological grade, Microvascular, Magnetic resonance imaging

## Abstract

**Background:**

To predict the histological grade and microvascular invasion (MVI) in patients with HCC.

**Methods:**

A retrospective analysis was conducted on 175 patients who underwent MRI enhancement scanning (from September 2016.9 to October 2020). They were divided into MVI positive, MVI negative, Grade-high and Grade-low groups.

**Results:**

The AFP of 175 HCC patients distributed in MVI positive and negative groups, Grade-low and Grade-high groups were statistically significant (P = 0.002 and 0.03, respectively). Multiple HCC lesions were more common in MVI positive and Grade-high groups. Correspondingly, more single lesions were found in MVI negative and Grade-low groups (P = 0.005 and 0.019, respectively). Capsule on MRI was more common in MVI negative and Grade-high groups, and the difference was statistically significant (P = 0.02 and 0.011, respectively). There were statistical differences in the distribution of three MRI signs: artistic rim enhancement, artistic peripheral enhancement, and tumor margin between MVI positive and MVI negative groups (P = 0.001, < 0.001, and < 0.001, respectively). Tumor hypointensity on HBP was significantly different between MVI positive and negative groups (P < 0.001).

**Conclusions:**

Our research shows that preoperative enhanced imaging can be used to predict MVI and tumor differentiation grade of HCC. The prognosis of MVI**-**negative group was better than that of MVI-positive group.

## Background

The histopathological grade of hepatocellular carcinoma (HCC) is one of the most important factors affecting the disease-free survival time, recurrence, and metastasis of HCC [1]. Compared with well-differentiated or moderately-differentiated HCC, poorly-differentiated HCC has poorer prognosis, higher recurrence rate, and lower survival rate. Liver biopsy is the only method for pathological grading of liver cancer before treatment. However, because of the invasion, sampling error, tumor planting, and bleeding, the wide application of preoperative biopsy is limited [2, 3]. Using non-invasive imaging technique to safely and accurately assess the pathological grade of HCC before operation will help clinicians to develop the best treatment methods for patients, improve the prognosis of patients, and also help to evaluate the indications of liver transplantation to wisely utilize scarce liver sources.

Similarly, microvascular invasion (MVI) is an important factor in predicting recurrence after surgical resection or liver transplantation, especially in predicting early recurrence after HCC operation [4, 5]. It is important to screen imaging indexes that can predict MVI before operation.

In the literature, quantitative ultrasound image analysis is used to distinguish HCC from borderline lesions and to predict the histological grade and MVI of HCC [6]. In other studies, MRI radiomics [7], via machine learning-based radiomics [8], diffusion weighted imaging [9, 10], and other imaging techniques are used to predict the histological grade of HCC. There are also a large number of studies that use various imaging techniques to predict MVI of HCC. Tumor size, edge, edge enhancement of arterial tumor, peritumoral enhancement of arterial tumor, and low signal intensity of hepatobiliary tumor have been found to be helpful in independently predicting MVI [11–14]. Our previous research [15] showed that rim enhancement in the artistic phase and peritumoral hypointensity in the hepatobiliary phase were independent risk factors for microvascular invasion in patients with HCC. The purpose of this study is to predict the histological grade and MVI by combining clinical data, imaging signs, and laboratory examination results, since we didn’t do so in our previous study.

## Methods

### Clinical data

**Fig. 1:**
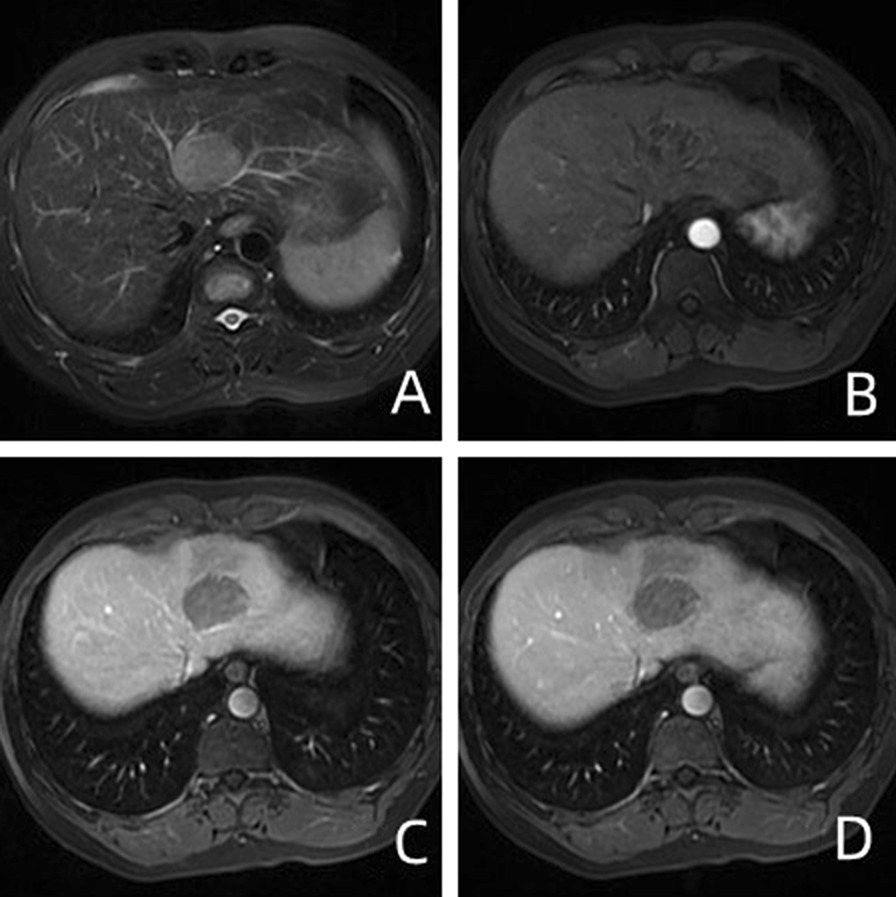
A 50-year-old man presented with low-grade HCC with MVI. **A** Unevenly high signals on T2WI; **B**, **C** Heterogeneous enhancement (black arrow) in the left lobe of liver in the arterial and delayed phases of GD-BOPTA–enhanced MRI; **D** Non-smooth tumor edge and peritumoral hypo-signal intensity (white arrow) in the hepatobiliary phase

A retrospective analysis was conducted on 175 patients who underwent MRI enhancement scanning between September 2016 and October 2020. The criteria for inclusion in this study are as follows: (1) The age is 18–80 years old, and surgical and pathological results are available; (2) Gd-BOPTA-enhanced MRI scan includes four phases: arterial phase, portal phase, delayed phase, and hepatobiliary phase; (3) MR examination has been completed within one week prior to the operation; (4) No other treatment, such as TACE and neoadjuvant chemotherapy, was received before MR examination (Fig. 1).

Clinical data and laboratory examination results were obtained from PACS (picture archiving and communication systems). The evaluation indexes include gender, age, alpha-fetoprotein (AFP), HBsAg, HBeAg, and liver cirrhosis.

This study was a retrospective study approved by ethics committees of our hospitals, and no informed consent was required.

### MRI scan

GE Application MR HDxt1.5 T, field strength 1.5 T and 8-channel abdominal surface coil were applied. Patients were fasted and refrained from drinking water for 4 h before scanning. Besides, patients were trained to breathe before scanning and to lie on the back on the examination bed with advanced feet. The contrast medium was injected into the median cubital vein with a high-pressure syringe at a flow rate of 2.0 ml/s using 0.1 mmoL/Kg meglumine gadoliniate injection. The enhanced scanning times were 22–25 s in arterial phase, 50–60 s in portal phase, and 90–120 s in delayed phase after injection of contrast medium. Transverse T1WI: gradient dual echo sequence was used, with breath held at the end of breath, TR/TE = 200/4.7 ms, slice thickness = 8 mm, slice interval = 2 mm, matrix = 228 × 160, FOV38 × 38 cm. Transverse T2WI: fast spin echo sequence, lipid pressing, and respiratory gating were performed, TR/TE = 12,000/85 ms, slice thickness = 5 mm, slice interval = 1 mm, matrix = 320 × 224, FOV = 38 × 38 × 0.75 cm. DWI:b = 5,0400,800 s/mm^2^. Respiratory gated scan: TR/TE = 13,000/67.9 ms, slice thickness = 5 mm, slice interval = 1 mm, matrix = 128 × 130, FOV = 38 × 38 cm. Enhancements: the 3-D LAVA technique was used in cross section, with TR/TE: 3.9/1.9, layer thickness: 4.8, matrix: 258 × 200, and FOV: 38 × 38 cm. During hepatobiliary period, coronal scan was added and the parameters were the same as those of enhanced scans.

### MRI signs analysis

The MR signs were evaluated by two radiologists, both of whom had more than 18 years of experience in liver imaging diagnosis. In case of doubt, they reached a consistent result after discussion. The evaluator had no prior knowledge about the clinical data of the patients.

**Fig. 2:**
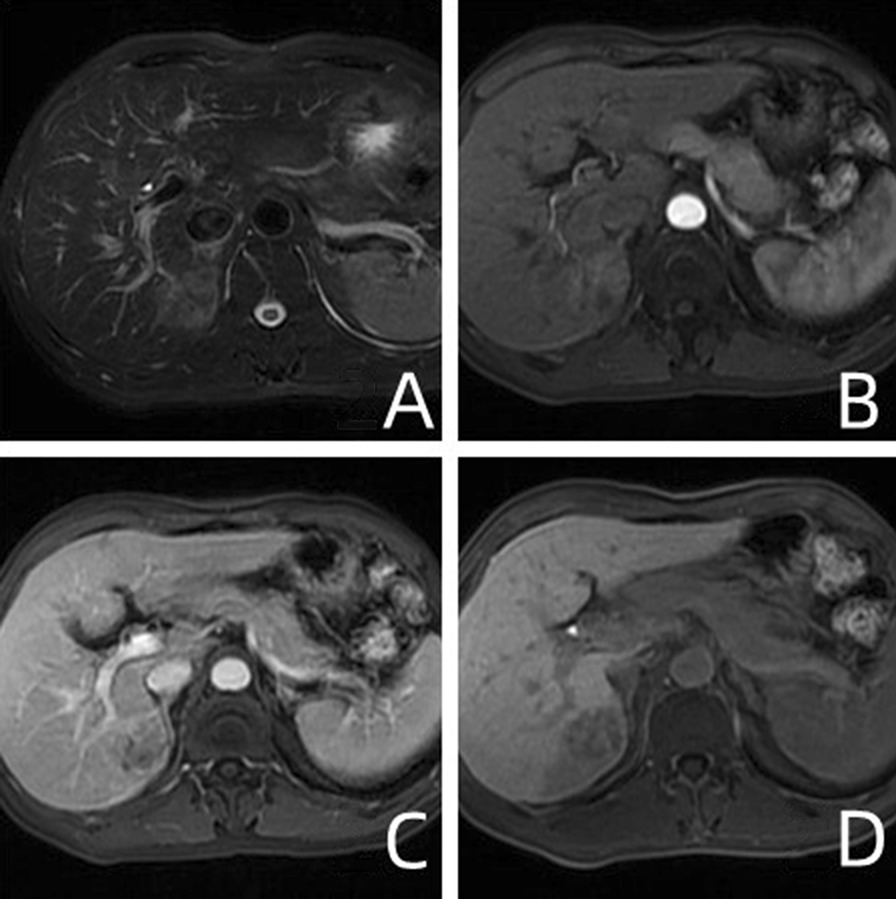
A 43-year-old male with High-grade HCC without MVI. **A** Unevenly high signals on T2WI; **B**, **C** In the arterial phase of GD-Bopta-enhanced MRI, the lesions showed obvious uneven enhancement, and the enhancement was weakened in the delayed phase. **D** The mass at the hepatobiliary phase has low signal intensity, with a peritumor area of low signal (white arrow)

Radiologists analyzed the diameter, margin, capsule, lipid composition, plain scan signal (Figs. 1A and 2A), arterial enhancement mode of enhanced scans (Figs. 1B, C, 2B, C), arterial peritumoral enhancement, hepatobiliary hyposignal, and peritumoral hyposignal (Figs. 1D and 2D), etc. According to the definition of LI-RADS-2017 [16] and previous literature [15], the longest diameter of the largest layer of the tumor including the capsule should be measured. For multiple lesions, we measured the diameter of the largest mass. Tumor margin is categorized into smooth and unsmooth. Intratumoral fat is the fat signal of more than 5% of the components in the mass. Envelope is defined as portal vein or smooth peripheral highly enhanced zone in delayed phase. Arterial peritumoral enhancement is a crescent-shaped or polygonal enhancement area beyond the edge of arterial tumor. The peritumoral hyposignal in hepatobiliary phase is defined as a wedge-shaped or flame-shaped hyposignal area beyond the edge of the hepatobiliary phase tumor.

### Analysis of pathological results

A pathologist who has been engaged in pathological diagnosis of liver tumors for 24 years was asked to review the pathological specimens of all cases. According to Edmondson's 4-grade classification method [17], the tumors were divided into I, II, III, and IV grades, with I–II, II–III, and III–IV grades in between. Our study used the classification of Ameli [3] for reference, and classified i, i–ii, and ii as low-grade tumors. Grade ii–iii, iii, iii–iv, and iv were classified as high-grade tumors.

Microvascular invasion (MVI): Cancer cell nests were observed in vascular lumen lined with endothelial cells under microscope. When the number of suspended cancer cells in vascular lumen was ≥ 50, it was considered as MVI. M0: no MVI was found; M1 (low risk group): ≤ 5 MVI, which occurred in liver tissue near the lesion; M2 (high-risk group): > 5 MVI, or MVI occurred in distant liver tissues [18]. After re-reading the histological specimens, we found that there were few M2 cases, so M1 and M2 were classified as MVI positive group and M0 as negative group.

### Statistical analysis

This study is divided into two groups, using the following statistical methods:

1. basic table: continuous variables (measurement data): they conform to normal distribution and are presented in the form of "mean +−sd" by t-test; non-normal distribution, using kruskal test, with "median (1/4–3/4 IQR)"; Classification variable (count/grade data): it is presented as "count (percentage)" by chi-square or Fisher test.
2. Univariate and multivariate analysis: Logistic regression. SPSS 19.0 statistical software package was used. Chi-square test and independent sample T test were used for statistical analysis, and the difference was statistically significant when P < 0.05.

## Results

### Clinical data and laboratory examination

See Table 1 for details. The AFP of 175 HCC patients distributed in MVI positive and negative groups, Grade-low and Grade-high groups were statistically significant (P = 0.002 and 0.03, respectively). The time of recurrence and metastasis was longer in MVI negative group than in MVI positive group (P = 0.012 and 0.017, respectively). There was also significant difference in liver cirrhosis between Grade-low group and Grade-high group (P = 0.011).

**Table 1:** Comparison of patient characteristics according to vascular invasion

Characteristics	Total	MVI			Grade		
		Negative	Positive	*P*	Low	High	*P*
Age	175	54.339 (10.584)	53.383 (10.742)	0.573	52.250 (10.606)	54.677 (10.587)	0.1780
Recurrence (months)	175	30.67 ± 23.43	22.18 ± 18.30	**0.012**	25.97 ± 20.67	21.72 ± 18.92	0.186
Metastasis (months)	175	30.08 ± 23.67	21.76 ± 18.96	**0.017**	26.03 ± 21.33	20.22 ± 18.79	0.077
HBsAg				0.080			0.590
Negative	26	21 (80.77)	5 (19.23)		6 (23.08)	20 (76.92)	
Positive	149	94 (63.09)	55 (36.91)		42 (28.19)	107 (71.81)	
HBeAg				0.706			0.266
Negative	117	78 (66.67)	39 (33.33)		29 (24.79)	88 (75.21)	
Positive	58	37 (63.79)	21 (36.21)		19 (32.76)	39 (67.24)	
Cirrhosis				0.522			**0.011**
Negative	46	32 (69.57)	14 (30.43)		6 (13.04)	40 (86.96)	
Positive	129	83 (64.34)	46 (35.66)		42 (32.56)	87 (67.44)	
AFP (ng/L)				**0.002**			**0.030**
> 0 < 20	81	63 (77.78)	18 (22.22)		30(37.04)	51 (62.96)	
> 20 < 400	58	36 (62.07)	22 (37.93)		11 (18.97)	47 (81.03)	
≥ 400	36	16 (44.44)	20 (55.56)		7 (19.44)	29 (80.56)	
*MRI feature*							
Tumor Number				**0.005**			**0.019**
Single	145	102 (70.34)	43 (29.66)		45 (31.03)	100 (68.97)	
Multiple	30	13 (43.33)	17 (56.67)		3 (10.00)	27 (90.00)	
Tumor size (cm)				**0.030**			0.979
< 5	131	92 (70.23)	39 (29.77)		36 (27.48)	95 (72.52)	
≥ 5	44	23 (52.27)	21 (47.73)		12 (27.27)	32 (72.73)	
Capsule				**0.020**			**0.011**
Negative	117	70 (59.83)	47 (40.17)		25 (21.37)	92 (78.63)	
Positive	58	45 (77.59)	13 (22.41)		23 (39.66)	35 (60.34)	
Lipid				0.609			0.078
Negative	121	81 (66.94)	40 (33.06)		38 (31.40)	83 (68.60)	
Positive	54	34 (62.96)	20 (37.04)		10 (18.52)	44 (81.48)	
Arterial rim enhancement				**0.001**			0.451
Negative	94	72 (76.60)	22 (23.40)		28 (29.79)	66 (70.21)	
Positive	81	43 (53.09)	38 (46.91)		20 (24.69)	61 (75.31)	
Arterial peritumoral enhancement				** < 0.001**			0.396
Negative	152	111 (73.03)	41 (26.97)		40 (26.32)	112 (73.68)	
Positive	23	4 (17.39)	19 (82.61)		8 (34.78)	15 (65.22)	
Tumor margin				** < 0.001**			0.883
Smooth	75	61 (81.33)	14 (18.67)		21 (28.00)	54 (72.00)	
Non-smooth	100	54 (54.00)	46 (46.00)		27 (27.00)	73 (73.00)	
Tumor hypointensity on HBP				0.291			0.177
Yes	161	104 (64.60)	57 (35.40)		42 (26.09)	119 (73.91)	
No	14	11 (78.57)	3 (21.43)		6 (42.86)	8 (57.14)	
Peritumoral hypointensity on HBP				** < 0.001**			0.178
Absent	146	106 (72.60)	40 (27.40)		43 (29.45)	103 (70.55)	
Present	29	9 (31.03)	20 (68.97)		5 (17.24)	24 (82.76)	
*Shape*				0.854			**0.044**
Round	58	40 (68.97)	18 (31.03)		23 (39.66)	35 (60.34)	
Oval	20	14 (70.00)	6 (30.00)		6 (30.00)	14 (70.00)	
Lobulated	65	41 (63.08)	24 (36.92)		11 (16.92)	54 (83.08)	
I rregular	32	20 (62.50)	12 (37.50)		8 (25.00)	24 (75.00)	

### Imaging signs

See Table 1 for details. There were significant differences in the number of tumors distributed in MVI positive and negative groups, Grade-low and Grade-high groups among 175 HCC patients (P = 0.005 and 0.019, respectively). Most lesions with diameter less than 5 cm were MVI negative, and the difference was statistically significant (P = 0.03). Capsule on MRI was more common in MVI negative group and Grade-high group, and the difference was statistically significant (P = 0.02 and 0.011, respectively). There were statistical differences in the distribution of three MRI signs: artistic rim enhancement, artistic peripheral enhancement, and tumor margin between MVI positive and MVI negative groups (P = 0.001, < 0.001, and < 0.001, respectively). The difference of HCC shape between Grade-low and Grade-high groups was statistically significant (P = 0.044).

### Logistic regression analysis

**Figs. 3–6:**
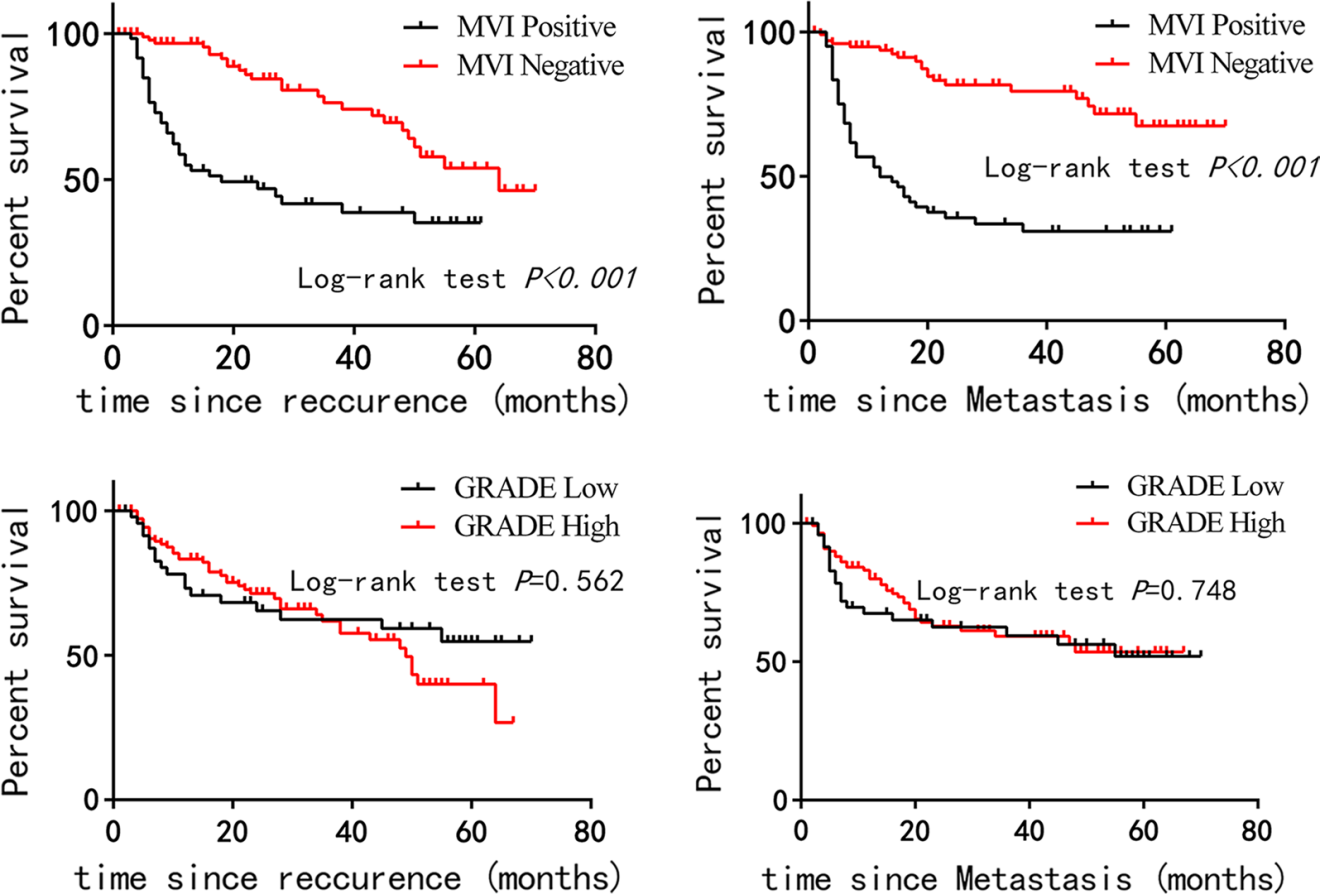
Survival analysis of recurrence and metastasis in MVI positive and negative groups (3–4), Grade-low and Grade-high groups (5–6)

Survival analysis of recurrence and metastasis in MVI positive group and negative group, Grade-low group and Grade-high group is shown in Figs. 3–6. The time of recurrence and metastasis in the MVI-negative group was longer than that in the MVI-positive group (P < 0.001).

Multivariate analysis showed that tumor size, tumor number, HBsAg, capsule, arterial peripheral enhancement, and tumor margin were independent risk factors for predicting MVI (Table 2). When these six indexes are used together, the specificity of MVI prediction is 100% (Table 3).

**Table 2:** Univariate and multivariate analyses of preoperative MR imaging findings in predicting MVI or Grade

	Univariate analysis (MVI)		multivariate analyses (MVI)		Univariate analysis (grade)		multivariate analyses (grade)	
	*OR* (95%*CI*)	*P*	*OR* (95%*CI*)	*P*	*OR* (95%*CI*)	*P*	*OR* (95%*CI*)	*P*
Age	0.991 (0.963, 1.021)	0.571			1.022 ( 0.990, 0.055)	0.178		
Sex	1.844 (0.812, 4.185)	0.143			0.520 (0.223, 1.210)	0.129		
Lipid	1.191 (0.61, 2.327)	0.609			2.014 (0.917, 4.424)	0.081	2.551 (0.96, 6.78)	0.06
Tumor Size	2.154 (1.069, 4.338)	0.032	5.174 (1.625, 16.477)	**0.005**	1.011 (0.47, 2.175)	0.979		
*AFP(ref: > 0, ≤ 20)*								
> 20, ≤ 400	2.139 (1.015, 4.508)	0.046	1.845 (0.729, 4.673)	0.196	2.513 (1.133, 5.574)	0.023	1.325 (0.529, 3.321)	0.548
> 400	4.375 (1.888, 10.14)	0.001	2.554 (0.84, 7.765)	0.098	2.437 (0.951, 6.242)	0.063	2.553 (0.804, 8.114)	0.112
HBsAg	2.457 (0.877, 6.887)	0.087	4.373 (1.193, 16.029)	**0.026**	0.764 (0.287, 2.036)	0.591		
HBeAg	1.135 (0.587, 2.194)	0.706			0.676 (0.339, 1.349)	0.267		
Tumor Number	3.102 (1.386, 6.94)	0.006	4.79 (1.565, 14.661)	**0.006**	4.05 (1.168, 14.046)	0.027	5.948 (1.345, 26.298)	**0.019**
Cirrhosis	1.267 (0.614, 2.613)	0.522			0.311 (0.122, 0.791)	0.014	0.252 (0.088, 0.719)	**0.010**
Capsule	0.43 (0.21, 0.883)	0.022	0.293 (0.098, 0.877)	**0.028**	0.414 (0.208, 0.822)	0.012	0.289 (0.123, 0.68)	**0.004**
Arterial rim enhancement	2.892 (1.515, 5.523)	0.001	2.141 (0.945, 4.853)	0.068	1.294 (0.661, 2.532)	0.452		
Arterial peritumoral enhancement	12.86 (4.129, 40.055)	< 0.001	5.02 (1.219, 20.68)	**0.026**	0.67 (0.264, 1.699)	0.398		
Tumor margin	3.712 (1.84, 7.485)	< 0.001	4.12 (1.621, 10.474)	**0.003**	1.052 (0.538, 2.055)	0.883		
Tumor hypointensity on HBP	0.498 (0.133, 1.857)	0.299			0.47 (0.154, 1.435)	0.185		
Peritumoral hypointensity on HBP	5.889 (2.475, 14.01)	< 0.001	1.898 (0.528, 6.829)	0.326	2.004 (0.717, 5.597)	0.185		
*Shape(ref: round)*								
Oval	0.952 (0.315, 2.879)	0.931			1.533 (0.515, 4.567)	0.443	3.08 (0.82, 11.572)	0.096
Lobulated	1.301 (0.614, 2.755)	0.492			3.226 (1.4, 7.435)	0.006	5.282 (1.962, 14.22)	**0.001**
Irregular	1.333 (0.539, 3.301)	0.534			1.971 (0.757, 5.136)	0.165	2.188 (0.71, 6.741)	0.173

**Table 3:** Diagnostic performance

Methods	Sensitivity	Specificity	Accuracy (%)	PPV (%)	NPV (%)
*MVI*					
Tumor size	35.00 (21/61)	80.00 (92/115)	64.57	47.73	70.23
HBsAg	91.67 (55/60)	18.26 (21/115)	43.43	36.91	80.77
Tumor Number	28.33 (17/60)	88.70 (102/115)	68.00	56.67	70.34
Capsule	78.33 (47/60)	39.13 (45/115)	52.57	40.17	77.59
Arterial peritumoral enhancement	31.67 (19/60)	96.52 (111/115)	74.29	82.61	73.03
Tumor margin	76.67 (46/60)	53.04 (61/115)	61.14	46.00	81.33
Series connection*	0.00 (0/60)	100.00 (115/115)	65.71	–	65.71
Parallel connection#	100.00 (60/60)	0.87 (1/115)	34.86	34.48	100.00
*Grade*					
Tumor Number	21.26 (27/127)	93.75 (45/48)	41.14	90.00	31.03
Cirrhosis	31.50 (40/127)	87.50 (42/48)	46.86	86.96	32.56
Capsule	72.44 (92/127)	47.92 (23/48)	65.71	78.63	39.66
shape	72.44 (92/127)	47.92 (23/48)	65.71	78.63	39.66
Series connection*	0.00 (0/127)	100.00 (48/48)	27.43	–	27.43
Parallel connection#	100.00 (127/127)	2.08 (1/48)	73.14	72.99	100.00

Tumor number, cirrhosis, capsule, and shape are independent risk factors for predicting high-grade HCC. When these four indexes are used together, the specificity of predicting high-grade HCC is 100% (Table 3).

## Discussion

The MVI of HCC is usually associated with later tumor stage and faster disease progression, the postoperative recurrence rate of MVI-positive patients is 4.4 times higher than that of MVI-negative patients [19]. A few years ago, the clinical significance of MVI was underestimated. It may be that MVI has been considered a slight manifestation of tumor invasion compared with great vessel infiltration [20]. Belonging to the scope of histopathological diagnosis, it is difficult to be identified by imaging examination. Histological classification of HCC is also an important predictor of recurrence and survival after hepatectomy and liver transplantation. Studying the correlation between imaging signs and histological classification can help clinicians choose appropriate preoperative treatment strategies. The postoperative survival rate of patients with well- and moderately differentiated HCC is significantly higher than that of patients with poorly differentiated HCC, and the 5-year postoperative recurrence rate of poorly differentiated HCC is as high as 70% [21]. For MVI-positive patients, the surgical resection area or radiofrequency ablation area must be expanded and systemic adjuvant therapy must be performed [22].

However, studies in recent years have shown that CT, MRI, and other imaging signs can be used to predict MVI to assist clinicians in developing treatment plans before operation, especially in choosing surgical plans. The existence of MVI will significantly reduce the survival rate after hepatectomy or liver transplantation, with the 3-year disease-free survival rates of patients with MVI and without MVI being 27.7% and 62.5%, respectively [23]. As the sample size and the variety of variables studied are different, it has been in dispute about which imaging signs can be used to stably and independently predict MVI.

Our results show that tumor diameter (> 5 cm), tumor number (multiple), HBsAg, Capsule (incomplete), arterial peritumoral enhancement, and tumor margin (unsmooth) are independent risk factors for predicting MVI. The multivariate analysis by Lei et al. [24] also showed that MVI was highly correlated with tumor diameter, multiple nodules, incomplete capsule, AFP greater than 20 ng/ml, platelet count and high DNA load of HBV. The reason of arterial peritumoral enhancement may be that in the peritumoral area with microvascular infiltration, tumor cells cause small portal vein branch occlusion, which leads to local portal vein blood flow reduction and compensatory arterial hyperperfusion [25]. It is reported in the literature [26–28] that the incidence of MVI in HCC with single nodule with exogenous and multiple nodule fusion is higher than that in HCC with smooth edges. This study also shows that the uneven edge of tumor can independently predict MVI.

There are many reports on predicting the histological grade of HCC using quantitative analysis techniques such as IVIM-DWI, conventional DWI, and DKI [3, 10, 29–31]. Most studies have proved the correlation between ADC value and histological grade of HCC, but some studies show that the difference of ADC value only exists between extreme groups (well differentiated HCC and poorly differentiated HCC), or it is concluded that there is no correlation between histological grade and ADC value [32]. In addition, in order to establish a reliable prediction model, it is necessary to use a low B value and a sufficient number of B values to obtain imaging. Yet, this actually limits the large-scale application in clinical practice, resulting in insufficient sample size and disputes over the selection of B values. In our study, clinical data, laboratory examination results, and MRI imaging signs were used to predict the histological grade of HCC, and it was found that Tumor Number (multiple), Cirrhosis, Capsule (incomplete), and Shape were independent risk factors for predicting high-grade HCC.

Our results showed that the time of recurrence and metastasis in the MVI-negative group was longer than that in the MVI-positive group, indicating that MVI-positive is an important factor affecting the prognosis of HCC patients. However, the prognostic difference between grade-low and grade-high groups was not statistically significant. We hope to collect more cases in the future to confirm that the grade of HCC can affect the recurrence, metastasis, and overall survival rate of patients.

The deficiency of this paper is that due to the retrospective analysis, HCC cases with DWI sequences were not sufficient for study, and the quantitative study for predicting MVI and histopathological grading was not done. In the next step, we will do some forward-looking design to study the relationship between IVIM-DWI parameters and MVI and the grade.

## Conclusions

Our research shows that preoperative enhanced imaging can be used to predict MVI and tumor differentiation grade of HCC. The prognosis of MVI-negative group was better than that of MVI-positive group.

## Data Availability

Data to replicate findings are in the Figures and Tables of the main paper. Due to patient privacy protection, any additional materials of the study are only available upon individual request directed to the corresponding author.

## Abbreviations

MVI: Microvascular invasion
HCC: Hepatocellular carcinoma
SI: Signal intensity
AFP: Alpha-fetoprotein
Gd-BOPTA: Gadobenate dimeglumine
DCE: Dynamic contrast enhanced
HBP: Hepatobiliary phase
